# Comparing the Effects of Sensory Tricks on Voice Symptoms in Patients With Laryngeal Dystonia and Essential Vocal Tremor

**DOI:** 10.1044/2024_JSLHR-24-00476

**Published:** 2025-02-27

**Authors:** Kaitlyn Dwenger, Nelson Roy, Skyler G. Jennings, Marshall E. Smith, Pamela Mathy, Kristina Simonyan, Julie M. Barkmeier-Kraemer

**Affiliations:** a Department of Communication Sciences and Disorders, The University of Utah, Salt Lake City; b Department of Otolaryngology–Head and Neck Surgery, The University of Utah, Salt Lake City; c Department of Otolaryngology–Head and Neck Surgery, Harvard Medical School and Massachusetts Eye and Ear, Boston; d Department of Neurology, Massachusetts General Hospital, Boston

## Abstract

**Purpose:**

This pilot study systematically compared voice symptomatology across varied sensory trick conditions in those with laryngeal dystonia (LD), those with essential vocal tremor (EVT), and vocally normal controls (NCs). Sensory tricks are considered signature characteristics of dystonia and were hypothesized to reduce voice symptoms in those with LD compared to EVT and NC groups.

**Method:**

Five participants from each group (LD, EVT, and NC) completed speech recordings under control and sensory trick conditions (delayed auditory feedback [DAF], vibrotactile stimulation [VTS], and nasoendoscopic recordings with and without topical anesthesia). Comparisons between groups and conditions were made using (a) a paired-comparison paradigm (control vs. sensory condition) listener ratings of voice quality, (b) participant-perceived vocal effort ratings, and (c) average smoothed cepstral peak prominence (CPPS).

**Results:**

Participants with EVT displayed significantly worse listener ratings under most sensory trick conditions, whereas participants with LD were rated significantly worse for DAF and VTS conditions only. However, participant vocal effort ratings were similar across all sensory trick conditions. Average CPPS values generally supported listener ratings across conditions and speakers except during DAF, wherein CPPS values increased (i.e., measurably improved voice quality), whereas listener ratings indicated worsened voice quality for both voice disorder groups.

**Conclusions:**

Outcomes of this study did not support the hypothesized influences of sensory trick conditions on LD voice symptoms, with both LD and EVT groups experiencing worsened symptoms under VTS and DAF conditions. These adverse effects on voice symptoms warrant further research to further evaluate neural pathways and associated sensorimotor response patterns that distinguish individuals with LD and EVT.

**Supplemental Material:**

https://doi.org/10.23641/asha.28462292

Focal laryngeal dystonia (LD) is a hyperkinetic movement disorder that consists of involuntary muscle contractions that are intermittent and unpredictable, affecting one body part, the larynx, with symptoms during volitional speech production (Albanese et al., 2013; Barkmeier et al., 2001; Ludlow et al., 2008, 2018; Simonyan et al., 2021). Historically, LD was referred to as spasmodic dysphonia (Aronson et al., 1968a, 1968b; Finitzo & Freeman, 1989; Murry, 2014; Woodson, 2005; Woodson et al., 1992). However, most recently, the term “laryngeal dystonia” was recommended with clinical phenotyping of the adductor or abductor types to align with movement disorder nomenclature and clinical characterization of this disorder (Blitzer et al., 2018; Simonyan et al., 2021). The more common adductor phenotype of LD is characterized by a strained–strangled voice quality with intermittent voice stoppages often corresponding with the production of voiced phonemes. The abductor clinical phenotype of LD is characterized by the onset of breathy voice breaks corresponding with voiceless-phoneme production (Barkmeier et al., 2001; Ludlow et al., 2008; Ludlow & Connor, 1987; Sapienza et al., 1999). LD is distinguished from other hyperkinetic movement disorders based on its task specificity. That is, symptoms are most commonly observed during the task of volitional speaking and less present during vegetative voicing (e.g., laughing, humming, throat clearing, and coughing) activities as well as during falsetto speaking or automatized speaking (e.g., counting) and reading where the cognitive load is lessened (Ludlow & Connor, 1987).

Essential tremor (ET) is an idiopathic movement disorder characterized by involuntary, bilateral action tremor affecting the upper extremities with a duration of 3 years or longer that can spread to involve tremor of other body parts (Bhatia et al., 2018; Lou & Jankovic, 1991; Louis et al., 2000; Merati et al., 2005). Tremor affecting speech structures in those with ET is referred to as essential vocal tremor (EVT; Bhatia et al., 2018). EVT is a neurogenic voice disorder characterized by a shaky or “quavering type of speech” and distinguished from other voice disorders by the perception of a rhythmic modulation of pitch and/or loudness during sustained vowel phonation (Merati et al., 2005; Sulica & Louis, 2010). However, there are inconsistencies within the literature regarding the clinical phenotype of EVT (Barkmeier-Kraemer, 2020; Merati et al., 2005; Torrecillas et al., 2021). The severity of EVT can be judged by the degree to which speech production is impaired (Lederle et al., 2012). In severe cases, EVT can be perceived during conversational speech and may exhibit similar clinical characteristics to LD, making differential diagnosis challenging for clinicians.

Differential diagnosis is essential for appropriate treatment planning and management of those with LD and EVT and for accurate clinical and epidemiologic characterization. Typically, a clinical diagnosis of a neurogenic voice disorder requires contributions from multiple disciplines consisting of experts in neurogenic voice, including the disciplines of speech-language pathology, otolaryngology, and neurology (Ludlow et al., 2018; Simonyan et al., 2021). Due to the paucity of clinicians with the necessary expertise to correctly diagnose individuals with LD, there is an average diagnosis delay of 5.5 years (de Lima Xavier & Simonyan, 2019). During this period, individuals report evaluation by an average of four clinicians before receiving their LD diagnosis (Creighton et al., 2015; Simonyan et al., 2021). Therefore, it is important to create readily accessible clinical tools to facilitate improved diagnostic precision between these neurogenic voice disorders that can be used by clinicians with varied levels of expertise. The recommended clinical voice assessment includes the completion of a thorough case history as well as systematic auditory-perceptual evaluation of the voice and visual-perceptual evaluation of the upper airway during imaging of a variety of vegetative and volitional speech tasks (Barkmeier-Kraemer & Clark, 2017; Roy et al., 2013; Simonyan et al., 2021; Stachler et al., 2018). Efforts to create consensus on the distinguishing characteristics of those with LD compared to those with vocal tremor and other voice disorders had mixed success (Ludlow et al., 2018). However, none of these approaches has been demonstrated to differentiate between EVT and LD with high classification accuracy or acceptable diagnostic precision. For example, the optimal reliability between expert raters representing multiple disciplines in distinguishing LD from other voice disorders, including vocal tremor, using laryngeal imaging and auditory-perceptual features is no greater than 34% agreement among experts (Ludlow et al., 2018). Furthermore, these prior studies did not assess one hallmark characteristic of dystonia during clinical testing—that is, “sensory tricks”—as a potential marker to improve differential diagnosis of patients with LD and EVT.

Sensory tricks or *geste antagoniste* is a phenomenon that occurs when a tactile or other proprioceptive maneuver ameliorates clinical symptomatology (Blitzer et al., 2018; LeDoux, 2012; N. Patel et al., 2014; Ramos et al., 2014). Variants of sensory tricks include tactile, visual, auditory, and thermal stimuli as well as imaginary, motor, and atypical tricks (e.g., forcible and reverse sensory tricks; LeDoux, 2012; N. Patel et al., 2014; Ramos et al., 2014). Literature on focal dystonias offers evidence to support that tactile sensory tricks ameliorate symptoms, specifically in those with cervical dystonia, focal hand dystonia, and dystonic limb tremor (Bending & Cleeves, 1990; Dagostino et al., 2019; Leis et al., 1992; Naumann et al., 2000; Pohl et al., 2002; Ramos et al., 2014; Tinazzi et al., 2005). In contrast, altered auditory feedback as an auditory type of sensory trick was deemed ineffective in a group with upper limb dystonia (Dagostino et al., 2019). The literature on sensory tricks in those with LD is primarily anecdotal without systematic testing of the contribution of various sensory modalities to changes in LD voice symptomatology. Based on the current literature, potential modalities to elicit sensory trick responses in those with LD include the presentation of a nasoendoscope, transcutaneous vibrotactile or electrical nerve stimulation, and auditory feedback conditions.

## Nasoendoscope Placement

During a typical voice assessment for dysphonia, individuals receive a competing tactile stimulus during the placement of a nasoendoscope through the nasal passageway into the pharynx. Furthermore, the administration of topical anesthesia during nasoendoscopic examination alters sensory feedback from the nasal and pharyngeal/laryngeal mucosa. Thus, the tactile stimuli of the scope itself or reduced sensation due to topical anesthesia can potentially ameliorate LD symptoms as a form of tactile sensory trick. Little has been published about this potential sensory trick effect from placing the nasoendoscope to the symptomatology classification of LD and other voice disorders (Ludlow et al., 2008, 2018; Stachler et al., 2018). However, early work by Ludlow et al. (2008) recommended that topical anesthesia not be used during nasoendoscopy to minimize such potential sensory trick contributions toward assessing speech features in those suspected of having LD. Therefore, it is important to consider the sensory trick phenomenon's contribution to clinical impressions during the various assessment methods clinicians currently use during an instrumental voice assessment.

## Vibrotactile Stimulation

The contribution of vibrotactile stimulation (VTS) toward improving the symptomatology of LD has been studied as a potential treatment modality (Khosravani et al., 2019). VTS includes a wide variety of gentle, vibratory sensations on the skin and body and has been shown to effectively reduce the severity of involuntary muscle contractions in other focal dystonias (Karnath et al., 2000; Leis et al., 1992). In a recent pilot feasibility study by Khosravani et al. (2019), VTS was shown to have a therapeutic effect on those with LD. Results showed that 69% of participants exhibited improved voice quality after an initial set of VTS (~15 min) and/or the second set of VTS (~20 min). Therefore, it shows promise as a sensory trick for short-term application during a clinical assessment and offers a noninvasive transcutaneous afferent stimulation similar to methods evaluated in other forms of dystonia (Leis et al., 1992; Tinazzi et al., 2005).

## Delayed Auditory Feedback

Another potential sensory feedback modality of a sensory trick associated with reduced symptomatic speech in those with LD is delayed auditory feedback (DAF). Ludlow et al. (1984) studied the effects of binaural presentation of 200-ms DAF on speech symptoms of patients with LD. One of the two patients who presented with voice symptoms consistent with LD and vocal tremor exhibited reduced symptoms while reading aloud during the DAF condition, whereas two patients with symptomatology consistent with LD without a vocal tremor did not show significant symptom changes using DAF (Ludlow et al., 1984). The clinical characteristics of the participants were also determined after the administration of topical anesthesia during the laryngoscopic examination, which may have altered symptomatology due to the competing tactile stimulus (i.e., a sensory trick) and confounded findings during the assessment. As such, there are mixed findings within the literature regarding the effects of DAF on the speech of those with LD. Therefore, further systematic testing of the effects of DAF and its potential for eliciting sensory trick responses in those with LD using a rigorous classification protocol is warranted to inform current clinical assessment practices.

The development of improved tools and methods for differential diagnosis of LD from other voice disorders was identified in 2008 and 2021 as a high research priority by experts representing multiple disciplines (Ludlow et al., 2008; Simonyan et al., 2021). Sensory tricks may offer a clinically useful and affordable diagnostic marker to differentiate individuals with LD from those with other voice disorders. This hallmark feature of dystonias has yet to be systematically tested in those with LD. Therefore, the purpose of this pilot study was to investigate the contribution of sensory tricks to changes in voice symptoms in individuals with LD compared to individuals with EVT and normal controls (NCs). Our hypotheses were that those with LD would exhibit significantly improved voice symptomatology under all or some of the sensory trick conditions tested, whereas voice symptoms in those with EVT and NCs would remain unchanged during all sensory trick conditions tested.

## Method

### Participants

#### Speakers

Five participants were recruited for each voice-disordered speaker group (LD and EVT). Five additional participants with normal voice quality were recruited as NCs who were age- and gender-matched to the voice-disordered speaker participants (±5 years of age). The specific inclusion criteria for all speaker participants are listed in Table 1. The LD group consisted of three men and two women with a median age of 67 years (interquartile range [IQR] = 27 years). The entire LD group presented with the adductor type of LD; however, one female participant presented with a co-occurring dystonic vocal tremor. The EVT group consisted of one man and four women with a median age of 71 years (IQR = 6 years). The NC group included three men and two women with a median age of 59 years (IQR = 32 years). Additional demographic and clinical characteristics, such as race and ethnicity as well as overall voice disorder severity, are summarized in Table 2, stratified by speaker group (LD, EVT, or NC).

**Table 1.** Inclusion criteria for all participants.

Speakers	
Voice disordered	18 years of age or older English as their primary communication language Normal hearing or no more than mild–moderate unilateral hearing loss Medical record diagnosis of LD (adductor type) or EVT A Voice Handicap Index questionnaire total score of 31 or more (moderate to severe) If being treated with botulinum toxin injections, last injection at least 3 months prior to participation If being treated with botulinum toxin injections, self-report a return of symptomatology to 50% or more of baseline symptoms (i.e., prior to starting botulinum toxin treatments) No neuromotor disorder other than dystonia or ET that affects their voice or speech No speech abnormalities due to stroke or traumatic brain injury No psychiatric disorder that may impact participation in the study
Normal controls	18 years of age or older English as their primary communication language Normal hearing or no more than mild–moderate unilateral hearing loss Normal voice quality No prior history of a voice disorder No prior history of a neuromotor or neurodegenerative disorder No known language or cognitive problems
**Listeners**	
	18 years of age or older English as their primary communication language Normal hearing or no more than mild–moderate unilateral hearing loss

*Note.* All participants were required to pass a pure-tone hearing screening using an audiometer presenting pure tones across the speech spectrum from 500 to 4000 Hz between 30 and 35 dB HL. LD = laryngeal dystonia; EVT = essential vocal tremor; ET = essential tremor.

**Table 2.** Demographic and clinical characteristics stratified by speaker participant group classification.

Characteristic	Expert group classification		
	LD (*N* = 5)	EVT (*N* = 5)	NC (*N* = 5)
Age (years) at study date, *Mdn* (IQR)	67 (27)	71 (6)	59 (32)
Sex, *n* (%)			
Female	2 (40)	4 (80)	2 (40)
Male	3 (60)	1 (20)	3 (60)
Race, *n* (%)			
White	5 (100)	4 (80)	5 (100)
Asian	0 (0)	1 (20)	0 (0)
African American	0 (0)	0 (0)	0 (0)
American Indian/Alaska Native	0 (0)	0 (0)	0 (0)
Native Hawaiian	0 (0)	0 (0)	0 (0)
Ethnicity, *n* (%)			
Hispanic or Latino	0 (0)	0 (0)	0 (0)
Non-Hispanic	5 (100)	5 (100)	5 (100)
Voice disorder severity, *n* (%)			
Mild	1 (20)	0 (0)	n/a
Mild–moderate	1 (20)	2 (40)	n/a
Moderate	1 (20)	0 (0)	n/a
Moderate–severe	1 (20)	2 (40)	n/a
Severe	1 (20)	1 (20)	n/a
Family history, *n* (%)			
Tremor	1 (20)	4 (80)	0 (0)
Dystonia	0 (0)	0 (0)	0 (0)
Comorbidities, *n* (%)			
Essential tremor	0 (0)	5 (100)	0 (0)
Cervical dystonia	1 (20)	0 (0)	0 (0)
Muscle tension dysphonia	2 (40)	0 (0)	0 (0)

*Note.* Chi-square and nonparametric Kruskal–Wallis tests revealed there were no significant differences between the three speaker groups for the above demographic and clinical characteristics, except for family history of tremor (*p* = .02) and a comorbidity of essential tremor (*p* = .001). These significant differences are expected and attributed to the EVT group having a greater percentage of a family history of tremor and comorbidity of essential tremor. LD = laryngeal dystonia; EVT = essential vocal tremor; NC = normal control; IQR = interquartile range; n/a = not applicable.

*Speaker clinical voice assessment*. Speaker participants (LD, EVT, or NC) meeting screening inclusion criteria subsequently underwent informed consent regarding the procedures for the study. All speaker participants completed a standard clinical voice assessment, including audio and nasoendoscopic recordings without topical anesthesia, immediately prior to the experimental audio recordings, as described below. A thorough case intake was completed to acquire basic demographic information as well as relevant medical and voice history. In addition, a cranial nerve tremor exam (Torrecillas et al., 2021) and pure-tone hearing screening were completed. Two expert clinicians representing speech-language pathology (J.M.B.K.) and laryngology (M.E.S.) with over 30 years of experience in diagnosing and managing voice disorders independently completed an auditory- and visual-perceptual analysis of each consented participant's audio and nasoendoscopic audiovisual recordings from the clinical voice assessment to confirm the classification of each speaker participant as LD, EVT, or NC using classification criteria as described in Appendix A. The operational definitions for each voice disorder were created using clinical features reported in the literature for LD (Barkmeier et al., 2001; Ludlow et al., 2008; Merati et al., 2005) and EVT (Bhatia et al., 2018; Merati et al., 2005). Each expert clinician marked the perceived and observed primary features for each speaker participant on the clinical classification form (see Appendix A) before ultimately classifying each participant as LD, EVT, or NC based on their clinical features. The expert clinicians were also asked to judge the overall degree of severity of the voice disorder of those classified with LD or EVT as a single judgment across the multiple contexts of speech from audio and nasoendoscopy recordings (see Appendix A). The goal was to include participants across the severity spectrum based on the participant's Voice Handicap Index (VHI) questionnaire total score. However, there was a focus on recruiting those rated as exhibiting moderate-to-severe symptomatology considering Khosravani et al. (2019) attributed nonresponsive participants to VTS as being more mild.

#### Listeners

Twelve naive listeners were recruited to complete auditory-perceptual judgments of paired-comparison audio recordings of the speaker participants collected during the experimental audio recording session described below. Inclusion criteria for the listener participants are also listed in Table 1.

### Experimental Procedure

The institutional review board approved all procedures conducted as part of this study at The University of Utah (IRB Protocol No. 00130977). The study session lasted approximately 2.5–3 hr and consisted of the clinical assessment procedures described above and experimental audio recordings under six separate conditions, including control and sensory trick conditions. The experimental audio recordings comprised the speaker participants completing a set of speech tasks, including sustained vowel and connected speech tasks, for each control or sensory trick condition (see Appendix B). The order and brief description of the six conditions are displayed in Table 3.

**Table 3.** Experimental condition order and abbreviations for the control and sensory trick conditions.

Condition order	Description	Abbreviation
1	Baseline control condition without additional sensory stimuli	Control 1
2	Placement of the nasoendoscope without administration of topical anesthesia (simultaneously with the clinical voice assessment nasoendoscopic recording tasks)	ENDO-A
3	Vibrotactile stimulation or delayed auditory feedback (alternated sequentially between participants within each group)	VTS/DAF
4	Vibrotactile stimulation or delayed auditory feedback (whichever condition was not selected above)	VTS/DAF
5	Second control condition without additional sensory stimuli	Control 2
6	Placement of the nasoendoscope after administration of topical anesthesia	ENDO+A

*Note.* Condition 2 was completed simultaneously with the clinical voice assessment nasoendoscopic examination recording tasks to minimize the number of times needed to pass the nasoendoscope. Conditions 3 and 4 were alternated between consecutive participants within each speaker group to control for order effects to the greatest degree possible. Condition 5 was completed to examine any adaptation effects from the repetition of the experimental speech tasks. Condition 6 was conducted last for all speaker participants due to the lingering effect of applying topical anesthesia for up to 1 hr to keep transition intervals between sensory conditions comparable.

#### Nasoendoscopy

The Pentax Medical Nasolaryngoscope System (KayPentax, Model 9310HD) was used to capture images of the soft palate, base of the tongue, pharyngeal walls, and larynx during the clinical voice assessment and experimental speech tasks. These images were obtained after the nasoendoscope (KayPentax, VNL-1070STK) was passed transnasally to the velopharynx where recordings of selected speech tasks were obtained for the clinical assessment prior to proceeding into the pharynx to view the larynx and oropharynx in entirety from the base of the tongue to the posterior pharyngeal wall for collection of the clinical assessment and experimental speech tasks. Two sensory conditions were used for study experimental recordings using nasoendoscopy: (a) placement of the nasoendoscope without administration of topical anesthesia (ENDO-A) during the voice assessment and (b) placement of the nasoendoscope after administration of topical anesthesia (ENDO+A). For the ENDO+A experimental condition, topical anesthesia was administered into the anterior nasal passage using a cotton swab dipped in viscous lidocaine (2%). Topical anesthesia was also administered transorally to ensure consistent dose and administration across participants. For the latter, a 30-cc plastic cup was used to mix 5 cc of viscous lidocaine (2%) and 5 cc of water at room temperature. All speaker participants were asked to gargle the mixture in their throat for as long as possible before swallowing it.

#### VTS

For the VTS sensory trick condition, a prototype of a laryngeal vibrotactile device developed by Passy Muir, Inc. in conjunction with Drs. Mulheren and Ludlow was used to apply VTS (Mulheren & Ludlow, 2017). This device includes two small flat motors overlying both sides of the thyroid lamina. In the present study, the device was secured using a soft, adjustable strap that wraps around the neck to deliver cutaneous vibratory stimulation to the larynx at 70 Hz. The rationale for employing a 70-Hz stimulation rate compared to 100 Hz as used in Khosravani et al.'s (2019) study was based on (a) the study by Mulheren and Ludlow (2017) and (b) reports in the motor rehabilitation literature associating muscle relaxation with lower frequencies (20–50 Hz) and increased muscle contraction at higher frequencies (100–150 Hz; Poenaru et al., 2016). Mulheren and Ludlow evaluated VTS administration across a range of stimulation rates from 30 to 150 Hz and determined that 70 and 150 Hz were optimal rates for activating laryngeal mechanoreceptors that trigger swallowing. In contrast to treatment applications of VTS, the current study evaluated the contribution of VTS to voice symptomatology as a sensory trick, defined as immediate changes to symptomatology upon application. As such, a short application of about 7 min of VTS, similar to one block of VTS administration used in Khosravani et al., was delivered while the participant was in the upright sitting position before recording the experimental speech tasks in the present study. An audio recording of the participant completing the speech tasks was acquired immediately upon onset of VTS administration followed by another audio recording completed after 5 min of VTS administration, for a total of 7 min of VTS. The two recordings under VTS allowed for comparisons regarding an immediate versus short-term dose effect of VTS.

#### DAF

For the DAF sensory trick condition, each speaker participant wore over-the-ear headphones (Sennheiser, Model HD 429) connected to the Computerized Speech Lab hardware associated with the Visi-Pitch software program containing the DAF output. During the DAF condition, the participant received bilateral playback of their voice via the headphones at a 150-ms delay from real time while speaking the selected speech stimuli. Comfortable loudness settings were determined by adjusting the playback gain levels for each participant to hear auditory feedback of their voice over their speaking voice ahead of simultaneous audio recordings of their voice.

#### Audio Recordings

Each participant's voice was recorded using a head-mounted AKG (Model C520) condenser microphone placed 45° off-axis to the mouth at a distance of ~5 cm (Švec & Granqvist, 2010, 2018). During signal acquisition, the microphone was connected to the Zoom H6 Pro handheld audio recorder, and all recordings were acquired at a 44.1-kHz sampling rate. Sound pressure level (SPL) measure calibration for voice recordings acquired using head-mounted microphones was conducted to enable comparable acoustic measures of voice intensity between speakers (Švec & Granqvist, 2010, 2018). This was achieved by using a Type 1 sound-level meter placed 30 cm from the front of the participant's mouth during a measurement of SPL for the background noise without the participant voicing, followed by a measurement of SPL while the participant sustained the vowel “ah” (Švec & Granqvist, 2018, p. 456). The gain settings of the microphone were first adjusted to ensure that the full dynamic range of intensity levels across speech tasks would be recorded without clipping the peaks of audio signals. Second, during calibration, it was noted whether each participant's recorded voice signal was at least 10 dB or greater above the ambient noise of the recording environment for optimal fidelity of SPL measures (Švec & Granqvist, 2010, 2018). Once calibration recording procedures were completed, speech tasks were recorded across all sensory trick conditions and control conditions using the identical gain settings for the duration of the experimental session.

The audio recordings for each speaker participant (LD, EVT, and NC) were segmented into individual speech tasks for each condition. See Appendix B for a list of the experimental speech tasks by speech stimuli type (i.e., sustained phonation and voiced-phoneme– or voiceless-phoneme–loaded sentences). The sustained phonation speech task audio recordings were segmented to include an audio file containing 2 s of sustained phonation of “ah” or “ee,” both taken from the midportion of each vowel. The connected speech task audio recordings were edited into separate files for each sentence. All audio files were deidentified and assigned a code number for blinded presentation to the listener participants.

#### Listener Ratings

**Figure 1.**
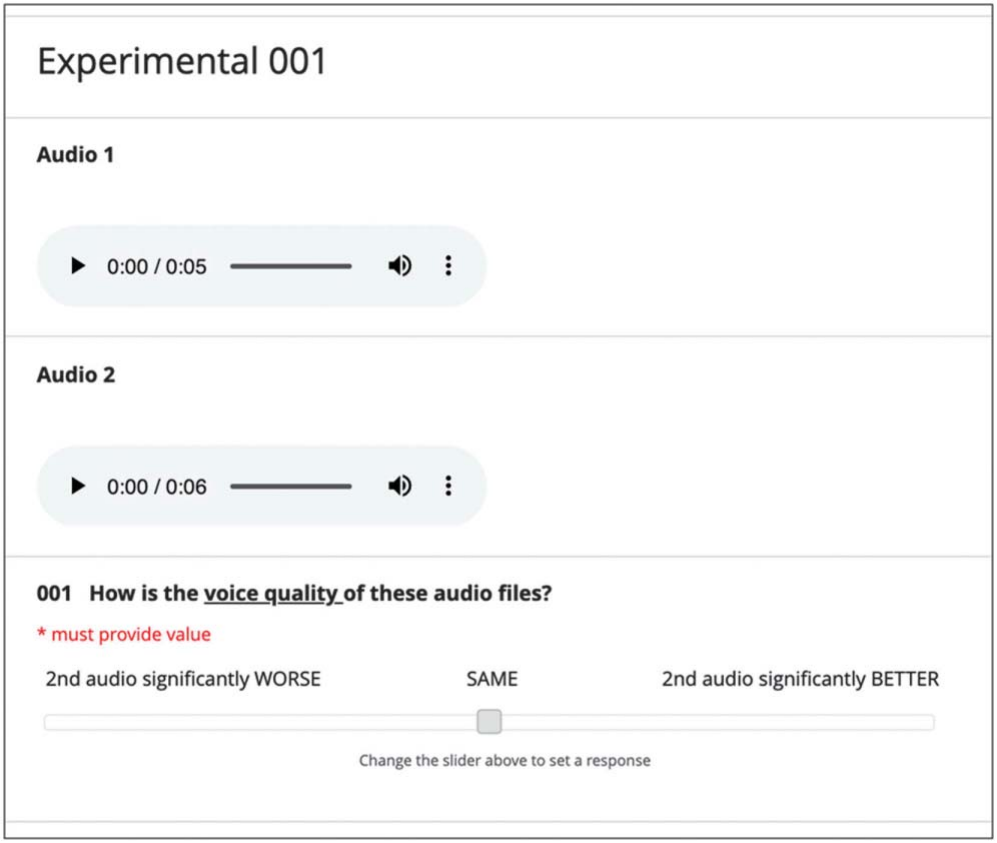
Example of the listener rating form for paired-comparison audio files.

For our primary measurement of outcomes, listeners completed independent listening and rating tasks of the overall voice quality of the speaker participants, including familiarization training to the rating task. A paired-comparison paradigm of the speaker participant speech task audio files was used for the listener ratings. As such, for each speaker participant, segmented speech task audio files from the first control condition (Condition 1) were used as the standard referent for comparison with the same segmented speech task audio file obtained during each of the sensory trick conditions (Conditions 2, 3, 4, or 6) or the second control condition (Condition 5). The order of paired comparisons of the speaker participant audio files were randomized, along with the order in which each individual audio file was played first within the pair. The audio files from the speaker participants were displayed using Research Electronic Data Capture software and played binaurally through over-the-ear headphones (KOSS, Model UR20) in a sound booth to the listeners at self-determined comfortable listening levels and rating pace. After listening to both audio files within the pair, listeners were asked to determine the degree to which the audio files were similar or dissimilar in terms of overall voice quality on a visual analog scale ranging from −100, indicating the second audio file had a significantly worse voice quality compared to the first audio file, to +100, indicating the second audio file had a significantly better voice quality compared to the first audio file (see Figure 1). The direct center of the visual analog scale represented a score of 0, meaning the listener judged the voice quality to be the same for the pair of audio files. The listeners were blinded to each condition during the rating task. To orient the listeners to the rating task and a range of auditory-perceptual features, they first listened to sample audio recordings of symptomatic voices from a different set of five speakers representative of the two voice-disordered groups but not included in the experimental task. Once oriented to the rating task and range of voices, the listeners began the listening experimental task.

Fifteen percent of the selected paired audio files were repeated to estimate intrarater reliability, and three versions of completely randomized listener rating lists were created to control for order effects of paired audio sequences across listeners. Thus, one of the three rating lists was randomly assigned to a listener who completed ratings on a total of 690 paired comparisons: 15 speaker participants × 5 condition pairing types (i.e., Condition 1 vs. 2, Condition 1 vs. 3, …, Condition 1 vs. 6) × 8 speech tasks (2 vowels and 6 sentences; see Appendix B) + 15% reliability ratings. To mitigate listener fatigue, breaks were given every 15 min or at any time needed, and listening sessions lasted no more than 1 hr for three to four total listening sessions, depending on the listener's rating pace. Listeners were compensated for their time monetarily or by university course credit.

#### Acoustic Measures

The secondary outcome measure for this study was a measure of the average smoothed cepstral peak prominence (CPPS) for each sustained vowel and connected speech sample to determine the overall voice quality. The CPPS was measured using Praat software (Version 6.1.38) and the parameters described by Murton et al. (2020). The CPPS is the recommended acoustic measure of overall voice quality (R. R. Patel et al., 2018) and is a reliable measurement for both vowels and connected speech (Awan et al., 2010; Maryn et al., 2009; Murton et al., 2020; Sauder et al., 2017).

#### Vocal Effort Ratings

**Figure 2.**
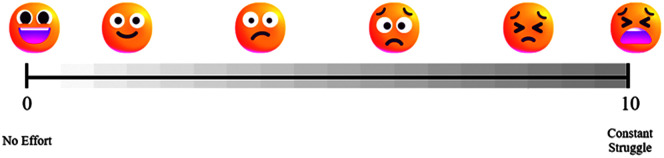
Vocal effort rating scale.

A measure of patient-perceived vocal effort was acquired immediately after speech recordings related to each experimental condition. The participants first rated their perceived vocal effort under the first baseline condition (Condition 1). That is, participants were asked to give a single rating of their speaking effort across all speaking tasks on a custom mixed-type scale, combining a Likert-type scale and a visual analog rating scale (see Figure 2). Then, this value was used as a standard referent by which the participants judged their perceived vocal effort on all subsequent conditions (Conditions 2–6).

### Statistical Analyses

Mixed-effects multiple linear regression models were used for the listener ratings and average CPPS acoustic measure dependent variables. The independent variables included in these models as fixed effects were group (NC, LD, and EVT) and condition (Control 1, ENDO-A, immediate application of VTS, DAF, Control 2, and ENDO+A). The individual listeners (listener ID), speaker participants (subject ID), and speech stimuli type (sustained phonation and voiced-phoneme– or voiceless-phoneme–loaded sentences) were included as random intercepts within the models to account for the correlation between listeners, participants, and speech stimuli type in the multilevel nested data structure. Two-way interactions of these fixed effects were examined first, followed by main effects. Post hoc analyses were conducted for the significant fixed effects using pairwise comparisons across all levels of the independent variables with Bonferroni's adjustment for multiple comparisons. The intraclass correlation (ICC) statistic was used to analyze interrater and intrarater reliability for the 12 naive listeners (Shrout & Fleiss, 1979). For the vocal effort ratings, nonparametric tests using the Kruskal–Wallis equality-of-populations rank test were used to determine if meaningful differences existed between groups and conditions within each group, followed by post hoc testing using Wilcoxon rank-sum (Mann–Whitney *U*) tests adjusted for multiple comparisons. A *p* value ≤ .05 was considered statistically significant for all comparisons. All data were analyzed using Stata statistical software (Version 17.0).

## Results

### Listener Rating Outcomes

Before conducting regression analyses for the naive listener ratings, intrarater and interrater reliability was examined. Five listeners demonstrated “good-to-excellent” intrarater reliability (ICC = .75–.90). Two listeners demonstrated “fair-to-moderate” agreement (ICC = .41–.74), and five listeners demonstrated “poor” agreement (ICC < .41). A cutoff value for the ICC statistic of .41 was used for the inclusion of listener ratings within the final analyses; thus, five listeners demonstrating poor agreement were excluded from the final analyses. With the seven listeners demonstrating “fair-to-excellent” intrarater agreement, interrater agreement was considered excellent with an ICC score of .79 (95% CI [.72, .79], *p* < .001) when data from all groups (NC, EVT, and LD) were combined. Lower but moderate interrater reliability was observed for the NC group alone (ICC = .66, 95% CI [.57, .73], *p* < .001). Excellent interrater reliability was observed for the LD (ICC = .82, 95% CI [.77, .86], *p* < .001) and EVT (ICC = .81, 95% CI [.77, .85], *p* < .001) groups.

**Figure 3.**
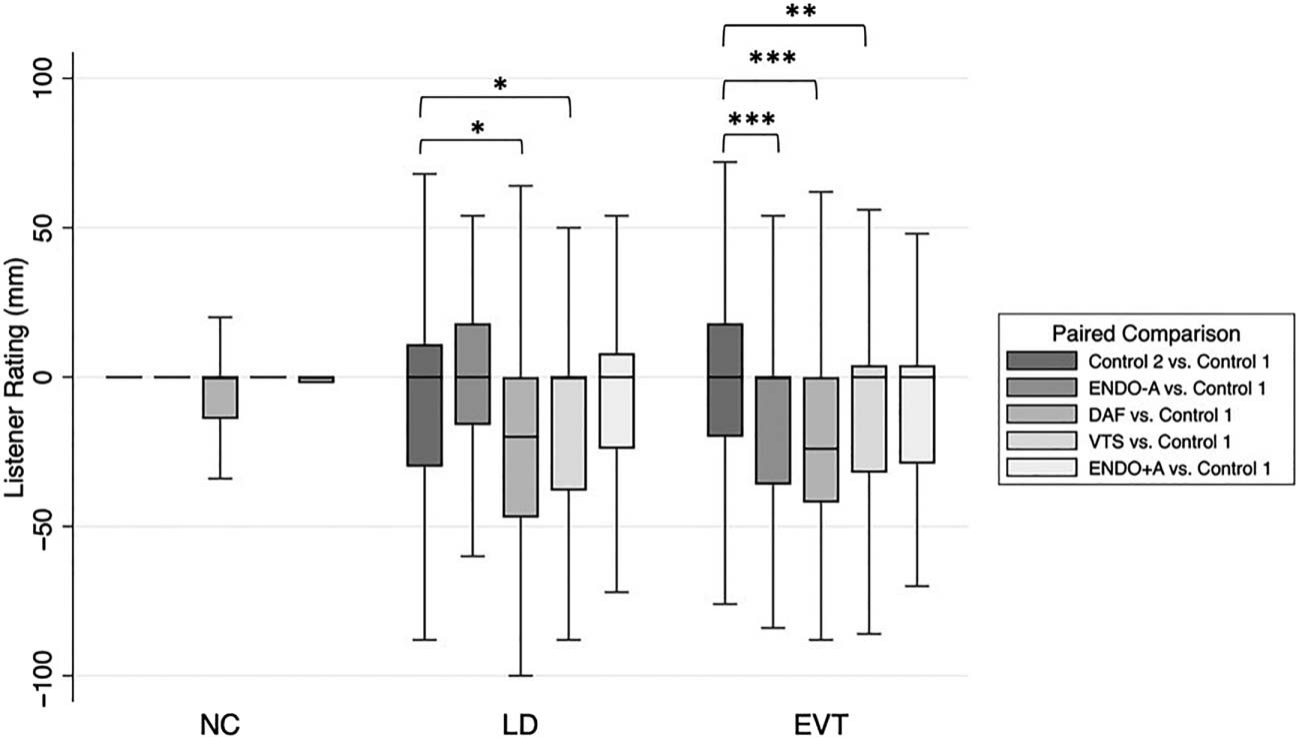
Listener ratings, the primary measurement of outcomes, within the paired-comparison paradigm, stratified by group and condition and aggregated across all speech stimuli types. The NC group was used as the standard referent for all comparisons. The asterisks indicate statistically significant differences between condition pairings and compared to the NC group as follows: *p ≤ .05, **p ≤ .01, and ***p ≤ .001. For example, compared to the NC group, the LD group had a statistically significant difference in listener ratings at the .05 level for the DAF condition paired comparison (i.e., when DAF and Control 1 recordings were compared together during listener rating tasks) compared to the Control 2 condition paired comparison (i.e., when Control 2 and Control 1 recordings were compared together during listener rating tasks). NC = normal control; LD = laryngeal dystonia; EVT = essential vocal tremor; DAF = delayed auditory feedback; VTS = vibrotactile stimulation; ENDO-A = placement of the nasoendoscope without administration of topical anesthesia; ENDO+A = placement of the nasoendoscope after administration of topical anesthesia.

Mixed-effects regression model estimates showed a significant interaction between speaker participant group and condition (*p*s ≤ .05) when controlling for the inherent variability between listener, speaker participant, and speech stimuli type. Specifically, model estimates showed that, compared to the regression model referents (i.e., the NC group and second control condition), the LD group received significantly decreased (i.e., worse) listener ratings for the DAF (*B* = −6.31, *SE* = 3.28, *p* = .05) and VTS (*B* = −6.99, *SE* = 3.28, *p* = .05) conditions. As illustrated in Figure 3 and Appendix C, listeners perceived the voice quality of those with LD to be worse under the DAF and VTS sensory trick conditions compared to the control condition. Compared to the referent conditions (i.e., NC group and second control condition), the EVT group also exhibited significantly decreased (i.e., worse) listener ratings for the DAF (*B* = −15.87, *SE* = 3.18, *p* < .001) and VTS (*B* = −8.68, *SE* = 3.20, *p* = .007) conditions as well as the ENDO-A condition (*B* = −12.94, *SE* = 3.19, *p* < .001; see Figure 3). The EVT group outcomes were found across all speech stimuli.

### Average CPPS Outcomes

**Figure 4.**
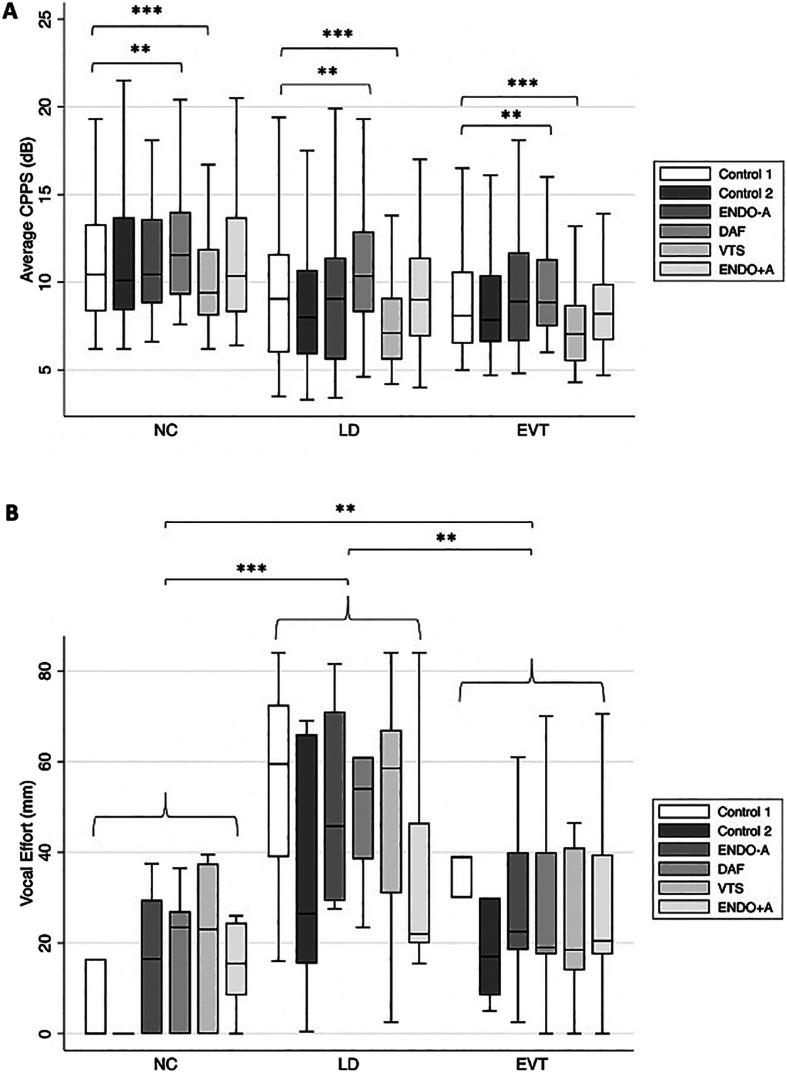
Average CPPS measures (A) and vocal effort ratings (B), secondary measurements of outcomes, stratified by group and condition and aggregated across all speech stimuli types. The asterisks indicate statistically significant differences between condition or group pairings as follows: **p ≤ .01, and ***p ≤ .001. CPPS = smoothed cepstral peak prominence; NC = normal control; LD = laryngeal dystonia; EVT = essential vocal tremor; ENDO-A = placement of the nasoendoscope without administration of topical anesthesia; DAF = delayed auditory feedback; VTS = vibrotactile stimulation; ENDO+A = placement of the nasoendoscope after administration of topical anesthesia.

The mixed-effects regression model estimates for the average CPPS values showed no significant interaction between speaker participant group and condition (*p*s ≥ .05) when controlling for the inherent variability between speaker participant and speech stimuli type. However, there was a significant main effect of condition (*p*s ≤ .01). Specifically, there was a statistically significant increase (improvement) in average CPPS values for the DAF condition (*B* = 0.73, *SE* = 0.27, *p* = .006) and a significant decrease (worsening) in average CPPS values for the VTS condition (*B* = −1.09, *SE* = 0.27, *p* < .001) compared to the baseline control condition regardless of speaker group. As illustrated in Figure 4A, higher (improved) average CPPS values were found for the DAF condition compared to the baseline control condition (Control 1) for all groups. This suggests that the DAF condition resulted in measurably improved overall voice quality for all speaker participants using average CPPS. In contrast, the VTS condition resulted in lower average CPPS values (worse voice quality) compared to the baseline control condition (Control 1) for all speaker participants (see Figure 4A). No significant differences in average CPPS were found between the baseline control condition and the two nasoendoscope conditions (ENDO-A and ENDO+A) or the second control condition across speaker participant groups. The main effect of the group was determined to not be statistically significant at the .05 significance level (*p* = .1).

### Vocal Effort Outcomes

The Kruskal–Wallis rank test for equality of populations revealed a statistically significant difference in vocal effort ratings between the groups (χ^2^ = 29.79, *p* < .001). Post hoc pairwise comparisons showed a statistically significant mean difference between the NC and LD groups (*z* = −5.23, *p* < .001), between the NC and EVT groups (*z* = −2.84, *p* = .005), and between the LD and EVT groups (*z* = 2.89, *p* = .004). Figure 4B shows that the NC group had lower vocal effort ratings than the LD and EVT groups across all conditions, whereas the vocal effort ratings were higher (worse) for the LD group compared to the EVT group across all conditions. Therefore, regardless of condition, the LD group had the highest (worst) vocal effort ratings compared to the EVT and NC groups. The Wilcoxon matched-pairs signed-ranks test revealed no statistically significant differences for any conditions within each speaker participant group. This nonsignificant finding may have resulted from the vocal effort ratings collected as a global rating across all speech stimuli for each condition (i.e., fewer observations of vocal effort ratings were collected compared to the other dependent variables). Additional descriptive tables for all outcome measures (i.e., listener ratings, average CPPS measures, and participant self-reported vocal effort ratings) have been made available in Supplemental Material S1–S3 for future comparisons to the statistically significant findings reported in this study.

## Discussion

The goal of this pilot study was to determine if systematic manipulation of sensory tricks as a distinguishing characteristic of dystonia resulted in significantly changed voice symptoms in individuals with LD compared to individuals with EVT and NCs. To achieve this goal, we examined the effects of DAF, VTS, ENDO+A, and ENDO-A as compared to no sensory manipulation (i.e., control conditions). The primary outcome measure was auditory-perceptual paired-comparison ratings of control versus experimental conditions by blinded naive listeners. In addition, the participants' self-ratings of vocal effort and acoustic measurement of voice quality using the average CPPS were compared across sensory trick and control conditions.

Prior published literature motivated the selection of the specific sensory conditions examined in this study (Khosravani et al., 2019; Ludlow et al., 1984, 2008; Mulheren & Ludlow, 2017). This literature reported mixed findings primarily described within case-based studies and largely anecdotal findings in individuals whose clinical features and diagnosis were not fully characterized as LD, EVT, or both. In addition, the determination of effects in this prior literature was based on single-clinician subjective ratings or outcome measures based on incompletely described experimental procedures, making replication challenging. Thus, the four sensory conditions identified in the literature were systematically assessed in this pilot controlled trial using within-subject and between-groups comparisons and a rigorous classification protocol for all participants.

On the basis of the extant literature describing sensory tricks in dystonia, we hypothesized that individuals with LD would exhibit significantly reduced voice symptomatology under all or some of the sensory trick conditions and that individuals with EVT and NCs would remain unchanged under all tested sensory trick conditions. The findings of this study did not support our hypotheses. We found a sensory effect in the LD and EVT groups as compared to the NC group. Furthermore, certain sensory tricks worsened symptoms in the groups with voice disorders, like that of a “reverse” sensory trick (Ramos et al., 2014). Although our results did not conform to our predictions, the outcomes provide important insights regarding the voice disorders studied.

### The Reverse Effect of VTS in LD

The VTS condition significantly worsened LD symptoms based on listener auditory-perceptual ratings of voice and CPPS measures. This finding was in contrast to prior published work by Khosravani et al. (2019), who examined VTS as a successful treatment method that reduces symptomatology in those with LD. The contrasting findings of our work compared to the published work using VTS for treating LD may be explained in part by the different methodologies between these studies. First, our VTS stimulation rate differed from this previously published work (Khosravani et al., 2019). VTS in this study was applied at a rate of 70 Hz, as published by Mulheren and Ludlow (2017), whereas Khosravani et al. administered VTS using a rate of 100 Hz. There is a possibility that the lower rate of VTS used in this present study activated different sensorimotor pathways than prior work using a higher rate. The 70-Hz stimulation rate was previously found optimal in activating mechanoreceptors that trigger swallowing (Mulheren & Ludlow, 2017). Thus, the 70 Hz used in this study may have activated a laryngeal protective response (laryngeal adduction) associated with an urge to swallow, resulting in increased strained–strangled voice quality in those with LD throughout speech production. In contrast, Khosravani et al. reported that VTS rates between 40 and 100 Hz were sufficient to stimulate mechanoreceptors and muscle spindles to evoke optimal motor responses. However, the literature in support of this finding is not based on laryngeal musculature (Bianconi & van der Meulen, 1963). Khosravani et al. found that 100 Hz elicited improved voice symptomatology as measured acoustically and cortically through event-related spectral perturbation measures. In contrast, Mulheren and Ludlow determined that a rate of 70- or 150-Hz, but not 110-Hz, stimulation was sufficient for activation of mechanoreceptors affecting laryngeal musculature through measured changes in fundamental frequency (*F*0) of the voice and cortical hemodynamic responses within the swallowing network (Mulheren & Ludlow, 2017).

A second difference between this study and the prior work using VTS for treating LD is the duration of stimulation and voice use during VTS application. Khosravani et al.'s (2019) group applied VTS during two identical sets of 8 min without vocalization, paired with adjacent sets of vocalizations of the vowel /a/ for 6.7 min. This was followed by voice assessment immediately after each of the two sets of VTS treatment. In the current study, VTS was administered for a total of 7 min, with voice recordings conducted immediately upon onset of VTS and after 5.5 min of administration while VTS remained on. No post-VTS speech assessment testing was conducted without VTS administration in the present study. The voice recordings made by this prior group were all conducted during reading rather than requiring speakers to repeat sentences. Our study used a combination of reading longer sentences and repeating shorter phrases as recommended by the recent multidisciplinary group publication (Simonyan et al., 2021). Khosravani et al.'s group measured symptom severity by recording the frequency of voice breaks and acoustic measures of CPPS. However, mixed VTS outcomes were reported across participants by severity. They attributed participants who had no effect of VTS to being milder without voice breaks. Our study only recruited those considered to have moderate-to-severe LD based on VHI total scores.

One last consideration regarding the methodology used for administering VTS is that our VTS device included straps around the neck to secure the device over the thyroid cartilage, in contrast to Khosravani et al.'s (2019) device, which was secured using skin adhesive. The VTS strap in our study could add another source of tactile sensation. However, this would be anticipated to enhance the VTS benefit rather than worsen voice symptoms in those with LD if it elicited a sensory trick response.

Another possible explanation for the unexpected difference in findings for VTS compared to Khosravani et al. (2019) relates to the proportion of participants reporting benefit from sensory tricks. Only one fifth (20%) of the participants with LD reported benefit from sensory tricks during case intake questionnaire procedures in this study; however, all five participants exhibited symptom changes from VTS based on listener ratings. It is reported in the literature that about 50% of patients with LD report symptom-based responses to sensory tricks (Blitzer et al., 2018). The report of presence or absence of sensory trick benefits by patients was not reported in the publication by Khosravani et al., but about 69% of cases were considered positive responders to VTS in their pilot feasibility study. Therefore, the participant-reported presence/absence of sensory tricks prior to testing may have influenced the difference in findings on VTS effects in patients with LD between these studies. The variability in a reported sensory trick response in LD is not unexpected given the heterogeneity in this patient population; however, it highlights the importance of examining whether a beneficial response to sensory manipulation via VTS only occurs in those with a specific clinical phenotype among those with dystonia in future studies.

To parse out the contribution of methodologic differences to outcomes of this pilot study and prior published work, future studies should systematically examine the effects of different rates of VTS on voice symptoms in those with LD. It has been reported that vibration frequency is a main factor in the effects of local vibratory stimulation on muscle and tendon junctions (Poenaru et al., 2016). Specifically, low frequencies of vibration (20–50 Hz) produce muscle relaxation, whereas the most widely used stimulation rates between 100 and 150 Hz alter the synchronization of motor units and elicit a vibratory reflex. In this phenomenon, vibration causes muscular contractions (Poenaru et al., 2016). Thus, future work should consider these varied rates of stimulation applied to the skin overlying the larynx to achieve intended changes in voice symptomatology in LD and EVT.

In addition to evaluating the symptom-related effects of VTS at different rates, cortical circuitry related to these different rates may be beneficial in elucidating the differences between our VTS outcomes and previous literature. Based on prior literature investigating VTS rates associated with triggering the swallow response, it is possible that our study triggered a brainstem reflexive pathway (Mulheren & Ludlow, 2017) that is present in all speaker groups rather than the cortical speech pathway previously shown to reduce LD symptoms (Khosravani et al., 2019). Therefore, evaluation of cortical responses at low- and high-frequency VTS rates should be conducted in future studies with these speaker groups to elucidate the underlying mechanism of worsened speech production under the VTS sensory condition in this study.

One last factor for the VTS condition that should be considered a potential contributing factor to naive listener ratings is an acoustic artifact in acoustic recordings measured between 71 and 77 Hz. Fortunately, this acoustic artifact was most perceptually evident during the nonvoiced breaks within connected speech samples and less evident during sustained phonation. Unfortunately, a method to filter this artifact from the audio signals was not achieved before conducting the listener ratings. Therefore, this artifact within the signals could have influenced the decrease in listener ratings for the VTS conditions. However, after completing their ratings, informal discussions with a subset of the listeners revealed that none reported hearing a “buzz” within any of the recordings. Furthermore, the effect of this artifact on the CPPS measures was less, given our technique of increasing the minimum frequency threshold from 60 to 80 Hz for computing the power cepstrogram in Praat. Comparison of CPPS measures with and without this adjustment to the power cepstrogram showed a 0.1- to 1-dB increase in average CPPS from measures without the adjustment across speech tasks, conditions, and groups. This raises the question of whether this small change in CPPS was perceptible by listeners as a change in voice quality. The threshold of CPPS values at which listeners correctly rate individuals with known voice disorders as having a voice disorder is a CPPS value below 14.5 dB for sustained phonation and 9.0 dB for connected speech (Murton et al., 2020). However, the minimum threshold of change between CPPS values required to perceptually detect a change in voice quality or severity is unknown. Therefore, it is possible that the measurable contribution of acoustic artifacts to CPPS in audio samples judged for VTS compared to the control condition accounted for the increased aberrance detected by listeners. However, future research is needed to determine the minimum detectable change in CPPS measures associated with a perceptible difference in voice quality to confirm that the acoustic artifact from the VTS influenced this study's findings.

Diagnostically, sensory manipulation using the vibrotactile device may have the potential to differentiate those with LD from those with other voice disorders. For example, past literature demonstrates that VTS can induce muscle relaxation at low frequencies of vibration (20–50 Hz; Poenaru et al., 2016). Thus, VTS may assist in amplifying symptoms of LD to clarify the presence and type of LD versus muscle tension dysphonia and/or vocal tremor. In this study, the VTS condition significantly worsened LD voice quality from the control condition. Thus, in a clinical context, if ambiguity surrounds a particular voice disorder diagnosis, VTS might be useful in provoking the underlying LD disorder to improve diagnostic clarity about the presence/absence and type of LD symptoms, particularly during connected speech tasks. Future work needs to be conducted to systematically test this hypothesis and determine whether VTS improves the contribution or degree of LD symptoms in those with other co-occurring symptoms before considering VTS (or sensory tricks) as a potential clinical tool to assist in the differential diagnosis of LD.

### Results for DAF in LD

This study demonstrated mixed results in those with LD for the DAF condition. The listener ratings indicated increased vocal aberrance for individuals with LD during DAF. In contrast, the CPPS measures reflected improved voice quality in those with LD. Interestingly, during the DAF condition, participants with LD perceptually altered their speaking patterns in an aberrant way, such as a slowed rate of speech, prolonged voicing, and increased loudness or pitch compared to the control condition without DAF. The NC group was used as a referent to determine if there were significant changes for the voice disorder groups compared to the NC group. However, they perceptually altered their speaking patterns similarly during DAF but without raters indicating perception of aberrant symptomatology. Thus, listener ratings of increased aberrance may have been less related to LD-specific symptom changes and, instead, reflected aberrance in the exaggerated speaking patterns resulting from the DAF condition. This finding is consistent with prior work on dysphonia in those with dysarthria characterized by hoarseness, increased strain, and pitch variability of the voice (Vogel et al., 2017). This prior work showed that speaking rate and syllable duration negatively impacted the listener's perception of the voice. In contrast, improvements in CPPS measures were found and attributed to the longer vowel productions in connected speech for this type of dysarthria (Vogel et al., 2017). To clarify the specific speaking features reflected by listener ratings in this present study, future work should replicate the listener rating protocol using expert listeners familiar with EVT and LD symptomatology to identify voice and speech features associated with DAF speech changes. In addition, speech parameters such as *F*0, SPL, and speech rate could be measured and compared associated with the DAF condition to identify covariates of listener rating and acoustic outcomes in this study.

The findings of this study align with prior research on DAF, which showed mixed outcomes (Daliri et al., 2020; Ludlow et al., 1984), although prior participants were poorly characterized. This study included well-defined groups through rigorous classification procedures and found a mixed effect of DAF for LD and EVT speaker groups. Therefore, speaking patterns associated with DAF may require additional consideration of the auditory–vocal reflex loop contributions to those with LD and EVT. Specifically, motor control of the voice is impacted by continuous monitoring of auditory feedback to control and correct perceived errors, similar to a negative feedback control system (Kim & Larson, 2019; Larson & Robin, 2016). Unexpected changes or perturbations of the voice based on auditory feedback result in compensatory changes in vocalizations (Kim & Larson, 2019; Larson & Robin, 2016). Therefore, DAF may disrupt or alter this response, causing the participants to change their speaking patterns as a compensatory strategy. However, the strategies used may have amplified the symptomatology of each speaker or their perception by listeners.

### Additional Voice Quality Changes in EVT

We hypothesized that those in the EVT group would not exhibit changes in speech symptoms under sensory trick conditions. Unexpectedly, the EVT group demonstrated significant changes in perceived voice ratings under most sensory conditions, suggesting there is also a sensory–motor component in EVT that has not been reported to date. Similar to the LD group, VTS was associated with greater voice aberrance in the EVT group. As with the LD group, it is possible that the rate of VTS stimulation activated a brainstem-level protective laryngeal adductory response resulting in increased laryngeal tension that would increase tremor in the affected muscles. Future research should test the hypothesis that VTS rate of stimulation originally designed to elicit a swallow response can negatively affect speech in those with EVT as well as those with LD. Similarly, a comparison to speech production during VTS at a slower rate of stimulation currently used for treating LD could be tested to determine any differences in those with LD compared to those with EVT in support of our original hypothesis.

Those with EVT also exhibited mixed results for the DAF condition. The listener ratings indicated more aberrant voicing for the DAF condition; however, average CPPS measures showed improved voice quality for the EVT group. Similar to the findings with the LD speaker group, it was unclear whether the outcomes for DAF in those with EVT reflected changes in vocal tremor severity or modified speaking patterns perceived by listeners as aberrant compared to the control condition. Repeating this study with expert listeners familiar with EVT speakers could clarify whether DAF was associated with changed vocal tremor symptoms or reflected changes in speaking patterns perceived as more aberrant by naive listeners. In addition, acoustic measures of *F*0, SPL, speech rate, and CPPS could be compared associated with listener percepts to determine significant covariates.

Prior research has not systematically assessed nasoendoscopy as a sensory trick; however, some experts contend that changes in voice symptoms may be observed in patients with LD during nasoendoscopic examination (Ludlow et al., 2008). Our pilot findings showed no statistically significant differences in voice symptoms under nasoendoscopic examination for the LD group. However, our listeners rated speech productions for the EVT group as more aberrant under the nasoendoscope condition without topical anesthesia, but no significant change in CPPS measures was associated with these ratings. Replicating this study with expert listeners might clarify whether changes observed by our naive listeners were related to changes in vocal symptoms specific to LD or EVT. A comparison of speaking patterns under the conditions of nasoendoscopy and the control condition with expert listeners would elucidate whether naive listeners detected an aberrant speaking pattern that reflected true changes in tremor symptomatology in those with EVT or different speaking patterns that were not necessarily associated with their voice disorder. Similarly, the use of expert listeners may help clarify whether naive listeners did not detect changes in intermittent voice stoppages in those with LD.

### Study Limitations

There are a few important limitations to note. This pilot study was conducted with a relatively small sample size, although the methodology for testing sensory trick effects used in this study resulted in a large number of observations for each speaker participant. One goal of this pilot study was to assess a small number of individuals meeting rigorous classification criteria, thereby controlling for the potential misdiagnosis of those with LD and EVT. Thus, on the basis of guidance from the literature on proof-of-concept pilot feasibility studies (Billingham et al., 2013; Leon et al., 2011; Moore et al., 2011), we aimed for a minimum of five participants in each group to inform future larger scale studies and population size estimates. An additional limitation for the LD group is the inclusion of those with adductor-type LD only. While the adductor type represents the largest phenotype of LD, findings from this study cannot be generalized to other subtypes of LD (Blitzer et al., 2018; Simonyan et al., 2021). Last, 42% of the listeners (*n* = 5) were excluded due to inadequate reliability levels. In addition, some of the listeners whose ratings were included in the analyses exhibited only fair-to-moderate reliability. Despite these limitations, interrater reliability on ratings for those with LD and EVT fell within the excellent range. Employing expert listeners might provide greater insight regarding specific types of symptom-based changes in the speaker groups studied; however, prior literature suggests they may not offer greater reliability in reporting specific clinical features across groups (Barkmeier et al., 2001; Eadie & Kapsner-Smith, 2011; Gerratt et al., 1993).

### Summary

This study was the first to examine the effects of sensory trick conditions on voice symptoms in those with LD versus those with EVT and NCs. Outcomes showed that VTS and DAF worsened the voice features in those with LD, whereas those with EVT demonstrated worsening of voice quality for the VTS, DAF, and ENDO-A sensory conditions. These findings were not anticipated and require further examination to clarify the specific influences of each sensory condition in LD versus EVT patterns. In general, this study's preliminary findings revealed that speakers meeting rigorous classification criteria for LD and EVT both demonstrated modified voice symptomatology under systematically administered sensory conditions. These results raise several compelling questions that require future research to better understand the clinical value of sensory conditions as a means to distinguish LD from other voice disorders. These questions should be further investigated prior to making conclusions regarding the clinical utility of sensory tricks to aid in the differential diagnosis of LD and EVT.

## Data Availability Statement

The data sets generated and analyzed during the current study are available from the corresponding author on reasonable request.

## Supplementary Material

10.1044/2024_JSLHR-24-00476SMS1 Supplemental Material S1 Descriptive statistics for the normal control group stratified by outcome measurement and condition.

10.1044/2024_JSLHR-24-00476SMS2 Supplemental Material S2 Descriptive statistics for the laryngeal dystonia group stratified by outcome measurement and condition.

10.1044/2024_JSLHR-24-00476SMS3 Supplemental Material S3 Descriptive statistics for the essential vocal tremor group stratified by outcome measurement and condition.

## Appendix A

### Clinical Classification Form by Which Experts Marked Primary Clinical Features and Overall Severity for Each Participant During the Clinical Consensus Participant Classification Procedures

**Table:**

Features					✓
**Laryngeal dystonia**	**Adductor type**	Auditory-perceptual	Intermittent (nonrhythmic occurrence) voice stoppages during production of voiced phonemes or sustained phonation		
			Intermittent onset of strained–strangled voice quality during sustained phonation or production of voiced phonemes		
			Symptoms most evident during connected speech; reduced or absent during sustained vowels		
			Symptoms reduced or absent during whispered speaking, crying, and laughing		
		Visual-perceptual	Normal structure and symmetry of the vocal folds at rest		
			Intermittent (not due to oscillatory) adductor spasms of laryngeal structures during connected speech associated with voiced phonemes		
		Case intake	Patient reports that sensory tricks lessen severity of symptoms.		
	**Abductor type**	Auditory-perceptual	Intermittent breathy breaks during production of voiceless phonemes		
			Symptoms most evident during connected speech; reduced or absent during sustained vowels		
		Visual-perceptual	Normal structure and symmetry of the vocal folds at rest		
			Intermittent (not due to oscillatory) abductor spasms of the laryngeal structures during connected speech associated with voiceless phonemes		
		Case intake	Patient reports that sensory tricks lessen severity of symptoms.		
**Muscle tension dysphonia**		Auditory-perceptual	Continuously strained voice quality across all speech tasks without intermittent changes to quality		
			Consistent aberrant voice quality without phoneme specificity		
			Symptoms are significantly reduced or eliminated with voice therapy stimulation probes.		
		Visual-perceptual	Continuous supraglottic compression of the laryngeal vestibule during speech		
**Vocal tremor**	**Dystonic**	Auditory-perceptual	Irregular rhythmic modulation of pitch and loudness during sustained phonation of vowels		
		Visual-perceptual	Tremor absent during respiration but present during volitional speech tasks (i.e., task specific)		
			Tremor mostly evident within the larynx (less visible or absent in other structures)	Soft palate	
				Pharyngeal walls	
				Larynx, vertical	
				Larynx, horizontal	
				Larynx/lengthwise	
				Base of tongue	
				Respiratory musculature	
		Case intake	Comorbidity of other forms of dystonia (cervical dystonia, blepharospasm) or diagnosis of focal dystonia of the larynx		
			Patient reports that sensory tricks lessen symptoms.		
	**Essential**	Auditory-perceptual	Nearly rhythmic modulation of pitch and loudness during sustained phonation of vowels		
			Voice stoppages or breathy breaks occur rhythmically with less evident phoneme specificity.		
		Visual-perceptual	Observation of tremor is not task specific (present during respiration and speaking).		
			Body distribution of tremor involves several sites (larynx, pharynx, tongue, soft palate) and possibly the respiratory musculature.	Soft palate	
				Pharyngeal walls	
				Larynx, vertical	
				Larynx, horizontal	
				Larynx/lengthwise	
				Base of tongue	
				Respiratory musculature	
		Case intake	Patient has a comorbidity of essential tremor.		
	**Parkinson's disease**	Auditory-perceptual	Rapid or irregular modulation of pitch and loudness during sustained phonation		
		Visual-perceptual	Tremor may appear irregular or inconsistently present during sustained phonation or speaking without phoneme specificity.		
			Body distribution of tremor may predominantly affect the larynx but may also involve other body structures (not well documented in the literature).	Soft palate	
				Pharyngeal walls	
				Larynx, vertical	
				Larynx, horizontal	
				Larynx/lengthwise	
				Base of tongue	
				Respiratory musculature	
		Case intake	Patient has a comorbidity of PD, or clinical features of bradykinesia/rigidity and asymmetric resting tremor including pill rolling tremor of hands.		
	**Isolated**	Auditory-perceptual	Nearly rhythmic modulation of pitch and loudness during sustained phonation of vowels		
			Voice stoppages or breathy breaks occur rhythmically with less evident phoneme specificity.		
		Visual-perceptual	Observation of tremor is not task specific (present during respiration and speaking).		
			Body distribution of tremor may involve several speech structures in the upper airway or respiratory musculature.	Soft palate	
				Pharyngeal walls	
				Larynx, vertical	
				Larynx, horizontal	
				Larynx/lengthwise	
				Base of tongue	
				Respiratory musculature	
		Case intake	Patient has no other neurological comorbidities.		
**Voice Quality Overall Severity**					
Mild			Trained listener would consider the voice abnormal; untrained listener would consider it unusual but normal. The characteristic is not distracting, and the ability to effectively communicate is not impaired.		
Moderate			Both trained and untrained listeners would consider the voice abnormal. The characteristic is distracting at times, and the ability to effectively communicate is noticeably impaired under certain circumstances.		
Severe			Both trained and untrained listeners would consider the voice extremely abnormal. The characteristic is highly distracting all the time, and the ability to effectively communicate is consistently affected.		

## Appendix B

### List of Speech Tasks Selected for the Experimental Procedures in This Study

1. Sustained vowel phonation:
  - Sustain /a/ at a comfortable pitch & loudness (~5 s)
  - Sustain /i/ at a comfortable pitch & loudness (~5 s)
2. Voiced-phoneme–loaded sentences:
  - Read: *Early one morning a man and a woman were ambling along a one-mile lane running near Rainy Island Avenue.**
  - Repeat after researcher:
    - The blue spot is on the key again. (4 corner vowel sentence)
    - We mow our lawn all year.
    - *We eat eggs every Easter.**
    - My mama makes lemon muffins.
    - *We were away a year ago.**
3. Voiceless-phoneme–loaded sentences:
  - Read: *He saw half a shape mystically cross a simple path at least fifty or sixty steps in front of his sister, Kathy's house.**
  - Repeat after researcher:
    - How hard did he hit him?
    - Peter will keep at the peak.
    - *The puppy bit the tape.**
    - Harry is happy because he has a new horse.
    - *Sally fell asleep in the soft chair.**

*Indicates the reduced set of sentences used for the paired-comparison listener ratings.

## Appendix C

### Listener Ratings Within the Paired-Comparison Paradigm for Each Subject in the LD Group Stratified by Ratings for the Control 2 and VTS Condition Pairings

*Note.* The average ratings were taken from the five listeners that demonstrated “good-to-excellent” intrarater reliability.

## References

[bib1] Albanese, A., Bhatia, K., Bressman, S. B., Delong, M. R., Fahn, S., Fung, V. S. C., Hallett, M., Jankovic, J., Jinnah, H. A., Klein, C., Lang, A. E., Mink, J. W., & Teller, J. K. (2013). Phenomenology and classification of dystonia: A consensus update. Movement Disorders, 28(7), 863–873. 10.1002/mds.25475 23649720 PMC3729880

[bib2] Aronson, A. E., Brown, J. R., Litin, E. M., & Pearson, J. S. (1968a). Spastic dysphonia. I. Voice, neurologic, and psychiatric aspects. Journal of Speech and Hearing Disorders, 33(3), 203–218. 10.1044/jshd.3303.203 4385966

[bib3] Aronson, A. E., Brown, J. R., Litin, E. M., & Pearson, J. S. (1968b). Spastic dysphonia. II. Comparison with essential (voice) tremor and other neurologic and psychogenic dysphonias. Journal of Speech and Hearing Disorders, 33(3), 219–231. 10.1044/jshd.3303.219 5668051

[bib5] Awan, S. N., Roy, N., Jetté, M. E., Meltzner, G. S., & Hillman, R. E. (2010). Quantifying dysphonia severity using a spectral/cepstral-based acoustic index: Comparisons with auditory-perceptual judgements from the CAPE-V. Clinical Linguistics & Phonetics, 24(9), 742–758. 10.3109/02699206.2010.492446 20687828

[bib8] Barkmeier, J. M., Case, J. L., & Ludlow, C. L. (2001). Identification of symptoms for spasmodic dysphonia and vocal tremor: A comparison of expert and nonexpert judges. Journal of Communication Disorders, 34(1–2), 21–37. 10.1016/S0021-9924(00)00039-3 11322567

[bib7] Barkmeier-Kraemer, J. M. (2020). Isolated voice tremor: A clinical variant of essential tremor or a distinct clinical phenotype? Tremor and Other Hyperkinetic Movements, 10, 1–8. 10.5334/tohm.535 32015933 PMC6988183

[bib6] Barkmeier-Kraemer, J. M., & Clark, H. M. (2017). Speech-language pathology evaluation and management of hyperkinetic disorders affecting speech and swallowing function. Tremor and Other Hyperkinetic Movements, 7, Article 489. 10.5334/tohm.381 PMC5628324 28983422

[bib9] Bending, J., & Cleeves, L. (1990). Effect of electrical nerve stimulation on dystonic tremor. The Lancet, 336(8727), 1385–1386. 10.1016/0140-6736(90)92946-F 1978199

[bib10] Bhatia, K. P., Bain, P., Bajaj, N., Elble, R. J., Hallett, M., Louis, E. D., Raethjen, J., Stamelou, M., Testa, C. M., & Deuschl, G. (2018). Consensus statement on the classification of tremors from the task force on tremor of the International Parkinson and Movement Disorder Society. Movement Disorders, 33(1), 75–87. 10.1002/mds.27121 29193359 PMC6530552

[bib11] Bianconi, R., & van der Meulen, J. (1963). The response to vibration of the end organs of mammalian muscle spindles. Journal of Neurophysiology, 26, 177–190. 10.1152/jn.1963.26.1.177 13968075

[bib12] Billingham, S. A., Whitehead, A. L., & Julious, S. A. (2013). An audit of sample sizes for pilot and feasibility trials being undertaken in the United Kingdom registered in the United Kingdom Clinical Research Network database. BMC Medical Research Methodology, 13, Article 104. 10.1186/1471-2288-13-104 PMC3765378 23961782

[bib13] Blitzer, A., Brin, M. F., Simonyan, K., Ozelius, L. J., & Frucht, S. J. (2018). Phenomenology, genetics, and CNS network abnormalities in laryngeal dystonia: A 30-year experience. The Laryngoscope, 128(Suppl. 1), S1–S9. 10.1002/lary.27003 PMC5757628 29219190

[bib14] Creighton, F. X., Hapner, E., Klein, A., Rosen, A., Jinnah, H. A., & Johns, M. M. (2015). Diagnostic delays in spasmodic dysphonia: A call for clinician education. Journal of Voice, 29(5), 592–594. 10.1016/j.jvoice.2013.10.022 25873547 PMC4868351

[bib15] Dagostino, S., Ercoli, T., Gigante, A. F., Pellicciari, R., Fadda, L., & Defazio, G. (2019). Sensory trick in upper limb dystonia. Parkinsonism & Related Disorders, 63, 221–223. 10.1016/j.parkreldis.2019.01.006 30655163

[bib58] Daliri, A., Heller Murray, E. S., Blood, A. J., Burns, J., Noordzij, J. P., Nieto-Castanon, A., Tourville, J. A., & Guenther, F. H. (2020). Auditory feedback control mechanisms do not contribute to cortical hyperactivity within the voice production network in adductor spasmodic dysphonia. Journal of Speech, Language, and Hearing Research, 63(2), 421–432. 10.1044/2019_JSLHR-19-00325 PMC7210444 32091959

[bib16] de Lima Xavier, L., & Simonyan, K. (2019). The extrinsic risk and its association with neural alterations in spasmodic dysphonia. Parkinsonism & Related Disorders, 65, 117–123. 10.1016/j.parkreldis.2019.05.034 31153765 PMC6774802

[bib17] Eadie, T. L., & Kapsner-Smith, M. (2011). The effect of listener experience and anchors on judgments of dysphonia. Journal of Speech, Language, and Hearing Research, 54(2), 430–447. 10.1044/1092-4388(2010/09-0205) 20884782

[bib18] Finitzo, T., & Freeman, F. (1989). Spasmodic dysphonia, whether and where: Results of seven years of research. Journal of Speech and Hearing Research, 32(3), 541–555. 10.1044/jshr.3203.541 2674551

[bib19] Gerratt, B. R., Kreiman, J., Antonanzas-Barroso, N., & Berke, G. S. (1993). Comparing internal and external standards in voice quality judgments. Journal of Speech and Hearing Research, 36(1), 14–20. 10.1044/jshr.3601.14 8450655

[bib20] Karnath, H. O., Konczak, J., & Dichgans, J. (2000). Effect of prolonged neck muscle vibration on lateral head tilt in severe spasmodic torticollis. Journal of Neurology, Neurosurgery, & Psychiatry, 69(5), 658–660. 10.1136/jnnp.69.5.658 11032623 PMC1763415

[bib21] Khosravani, S., Mahnan, A., Yeh, I. L., Aman, J. E., Watson, P. J., Zhang, Y., Goding, G., & Konczak, J. (2019). Laryngeal vibration as a non-invasive neuromodulation therapy for spasmodic dysphonia. Scientific Reports, 9(1), Article 17955. 10.1038/s41598-019-54396-4 PMC6884515 31784618

[bib22] Kim, J. H., & Larson, C. R. (2019). Modulation of auditory–vocal feedback control due to planned changes in voice *f*_o_. The Journal of the Acoustical Society of America, 145(3), 1482–1492. 10.1121/1.5094414 31067945 PMC6433561

[bib23] Larson, C. R., & Robin, D. A. (2016). Sensory processing: Advances in understanding structure and function of pitch-shifted auditory feedback in voice control. AIMS Neuroscience, 3(1), 22–39. 10.3934/Neuroscience.2016.1.22

[bib24] Lederle, A., Barkmeier-Kraemer, J., & Finnegan, E. (2012). Perception of vocal tremor during sustained phonation compared with sentence context. Journal of Voice, 26(5), 668.e1–668.e9. 10.1016/j.jvoice.2011.11.001 22521323

[bib25] LeDoux, M. S. (2012). Dystonia: Phenomenology. Parkinsonism & Related Disorders, 18(Suppl. 1), S162–S164. 10.1016/S1353-8020(11)70050-5 22166421 PMC4869992

[bib4] Leis, A. A., Dimitrijevic, M. R., Delapasse, J. S., & Sharkey, P. C. (1992). Modification of cervical dystonia by selective sensory stimulation. Journal of the Neurological Sciences, 110(1–2), 79–89. 10.1016/0022-510X(92)90013-B 1506873

[bib27] Leon, A. C., Davis, L. L., & Kraemer, H. C. (2011). The role and interpretation of pilot studies in clinical research. Journal of Psychiatric Research, 45(5), 626–629. 10.1016/j.jpsychires.2010.10.008 21035130 PMC3081994

[bib26] Lou, J. S., & Jankovic, J. (1991). Essential tremor: Clinical correlates in 350 patients. Neurology, 41(2, Pt. 1), 234–238. 10.1212/WNL.41.2_Part_1.234 1992367

[bib28] Louis, E. D., Ford, B., & Barnes, L. F. (2000). Clinical subtypes of essential tremor. Archives of Neurology, 57(8), 1194–1198. 10.1001/archneur.57.8.1194 10927801

[bib29] Ludlow, C. L., Adler, C. H., Berke, G. S., Bielamowicz, S. A., Blitzer, A., Bressman, S. B., Hallett, M., Jinnah, H. A., Juergens, U., Martin, S. B., Perlmutter, J. S., Sapienza, C., Singleton, A., Tanner, C. M., & Woodson, G. E. (2008). Research priorities in spasmodic dysphonia. Otolaryngology—Head and Neck Surgery, 139(4), 495–505. 10.1016/j.otohns.2008.05.624 18922334 PMC2643054

[bib30] Ludlow, C. L., & Connor, N. P. (1987). Dynamic aspects of phonatory control in spasmodic dysphonia. Journal of Speech and Hearing Research, 30(2), 197–206. 10.1044/jshr.3002.197 3599951

[bib31] Ludlow, C. L., Domangue, R., Sharma, D., Jinnah, H. A., Perlmutter, J. S., Berke, G., Sapienza, C., Smith, M. E., Blumin, J. H., Kalata, C. E., Blindauer, K., Johns, M., Hapner, E., Harmon, A., Paniello, R., Adler, C. H., Crujido, L., Lott, D. G., Bansberg, S. F., … Stebbins, G. (2018). Consensus-based attributes for identifying patients with spasmodic dysphonia and other voice disorders. JAMA Otolaryngology—Head & Neck Surgery, 144(8), 657–665. 10.1001/jamaoto.2018.0644 29931028 PMC6143004

[bib32] Ludlow, C. L., Naunton, R. F., & Bassich, C. J. (1984). Procedures for the selection of spastic dysphonia patients for recurrent laryngeal nerve section. Otolaryngology—Head and Neck Surgery, 92(1), 24–31. 10.1177/019459988409200105 6422412

[bib33] Maryn, Y., Dick, C., Vandenbruaene, C., Vauterin, T., & Jacobs, T. (2009). Spectral, cepstral, and multivariate exploration of tracheoesophageal voice quality in continuous speech and sustained vowels. The Laryngoscope, 119(12), 2384–2394. 10.1002/lary.20620 19718753

[bib34] Merati, A. L., Heman-Ackah, Y. D., Abaza, M., Altman, K. W., Sulica, L., & Belamowicz, S. (2005). Common movement disorders affecting the larynx: A report from the neurolaryngology committee of the AAO-HNS. Otolaryngology—Head and Neck Surgery, 133(5), 654–665. 10.1016/j.otohns.2005.05.003 16274788

[bib35] Moore, C. G., Carter, R. E., Nietert, P. J., & Stewart, P. W. (2011). Recommendations for planning pilot studies in clinical and translational research. Clinical and Translational Science, 4(5), 332–337. 10.1111/j.1752-8062.2011.00347.x 22029804 PMC3203750

[bib36] Mulheren, R. W., & Ludlow, C. L. (2017). Vibration over the larynx increases swallowing and cortical activation for swallowing. Journal of Neurophysiology, 118(3), 1698–1708. 10.1152/jn.00244.2017 28679839 PMC5596143

[bib37] Murry, T. (2014). Spasmodic dysphonia: Let's look at that again. Journal of Voice, 28(6), 694–699. 10.1016/j.jvoice.2014.03.007 24972536

[bib38] Murton, O., Hillman, R., & Mehta, D. (2020). Cepstral peak prominence values for clinical voice evaluation. American Journal of Speech-Language Pathology, 29(3), 1596–1607. 10.1044/2020_AJSLP-20-00001 32658592 PMC7893528

[bib39] Naumann, M., Magyar-Lehmann, S., Reiners, K., Erbguth, F., & Leenders, K. L. (2000). Sensory tricks in cervical dystonia: Perceptual dysbalance of parietal cortex modulates frontal motor programming. Annals of Neurology, 47(3), 322–328. 10.1002/1531-8249(200003)47:3<322::AID-ANA7>3.0.CO;2-E 10716251

[bib40] Patel, N., Hanfelt, J., Marsh, L., & Jankovic, J. (2014). Alleviating manoeuvres (sensory tricks) in cervical dystonia. Journal of Neurology, Neurosurgery, & Psychiatry, 85(8), 882–884. 10.1136/jnnp-2013-307316 24828895 PMC4871143

[bib41] Patel, R. R., Awan, S. N., Barkmeier-Kraemer, J., Courey, M., Deliyski, D., Eadie, T., Paul, D., Švec, J. G., & Hillman, R. (2018). Recommended protocols for instrumental assessment of voice: American Speech-Language-Hearing Association Expert Panel to Develop a Protocol for Instrumental Assessment of Vocal Function. American Journal of Speech-Language Pathology, 27(3), 887–905. 10.1044/2018_AJSLP-17-0009 29955816

[bib42] Poenaru, D., Cinteza, D., Petrusca, I., Cioc, L., & Dumitrascu, D. (2016). Local application of vibration in motor rehabilitation—Scientific and practical considerations. Maedica, 11(3), 227–231. 28694858 PMC5486165

[bib43] Pohl, C., Happe, J., & Klockgether, T. (2002). Cooling improves the writing performance of patients with writer's cramp. Movement Disorders, 17(6), 1341–1344. 10.1002/mds.10251 12465079

[bib44] Ramos, V. F., Karp, B. I., & Hallett, M. (2014). Tricks in dystonia: Ordering the complexity. Journal of Neurology, Neurosurgery, & Psychiatry, 85(9), 987–993. 10.1136/jnnp-2013-306971 24487380 PMC4747630

[bib45] Roy, N., Barkmeier-Kraemer, J., Eadie, T., Sivasankar, M. P., Mehta, D., Paul, D., & Hillman, R. (2013). Evidence-based clinical voice assessment: A systematic review. American Journal of Speech-Language Pathology, 22(2), 212–226. 10.1044/1058-0360(2012/12-0014) 23184134

[bib46] Sapienza, C. M., Walton, S., & Murry, T. (1999). Acoustic variations in adductor spasmodic dysphonia as a function of speech task. Journal of Speech, Language, and Hearing Research, 42(1), 127–140. 10.1044/jslhr.4201.127 10025549

[bib47] Sauder, C., Bretl, M., & Eadie, T. (2017). Predicting voice disorder status from smoothed measures of cepstral peak prominence using Praat and Analysis of Dysphonia in Speech and Voice (ADSV). Journal of Voice, 31(5), 557–566. 10.1016/j.jvoice.2017.01.006 28169094

[bib48] Shrout, P. E., & Fleiss, J. L. (1979). Intraclass correlations: Uses in assessing rater reliability. Psychological Bulletin, 86(2), 420–428. 10.1037/0033-2909.86.2.420 18839484

[bib49] Simonyan, K., Barkmeier-Kraemer, J., Blitzer, A., Hallett, M., Houde, J. F., Jacobson Kimberley, T., Ozelius, L. J., Pitman, M. J., Richardson, R. M., Sharma, N., & Tanner, K. (2021). Laryngeal dystonia: Multidisciplinary update on terminology, pathophysiology, and research priorities. Neurology, 96(21), 989–1001. 10.1212/WNL.0000000000011922 33858994 PMC8205448

[bib50] Stachler, R. J., Francis, D. O., Schwartz, S. R., Damask, C. C., Digoy, G. P., Krouse, H. J., McCoy, S. J., Ouellette, D. R., Patel, R. R., Reavis, C. C. W., Smith, L. J., Smith, M., Strode, S. W., Woo, P., & Nnacheta, L. C. (2018). Clinical practice guideline: Hoarseness (dysphonia) (update). Otolaryngology—Head and Neck Surgery, 158(Suppl. 1), S1–S42. 10.1177/0194599817751030 29494321

[bib51] Sulica, L., & Louis, E. D. (2010). Clinical characteristics of essential voice tremor: A study of 34 cases. The Laryngoscope, 120(3), 516–528. 10.1002/lary.20702 20066728

[bib52] Švec, J. G., & Granqvist, S. (2010). Guidelines for selecting microphones for human voice production research. American Journal of Speech-Language Pathology, 19(4), 356–368. 10.1044/1058-0360(2010/09-0091) 20601621

[bib53] Švec, J. G., & Granqvist, S. (2018). Tutorial and guidelines on measurement of sound pressure level in voice and speech. Journal of Speech, Language, and Hearing Research, 61(3), 441–461. 10.1044/2017_JSLHR-S-17-0095 29450495

[bib54] Tinazzi, M., Farina, S., Bhatia, K., Fiaschi, A., Moretto, G., Bertolasi, L., Zarattini, S., & Smania, N. (2005). TENS for the treatment of writer's cramp dystonia: A randomized, placebo-controlled study. Neurology, 64(11), 1946–1948. 10.1212/01.WNL.0000163851.70927.7E 15955950

[bib55] Torrecillas, V., Dwenger, K., & Barkmeier-Kraemer, J. M. (2021). Classification of vocal tremor using updated consensus-based tremor classification criteria. Laryngoscope Investigative Otolaryngology, 6(2), 261–276. 10.1002/lio2.544 33869758 PMC8035951

[bib56] Vogel, A. P., Wardrop, M. I., Folker, J. E., Synofzik, M., Corben, L. A., Delatycki, M. B., & Awan, S. N. (2017). Voice in Friedreich ataxia. Journal of Voice, 31(2), 243.e9–243.e19. 10.1016/j.jvoice.2016.04.015 27501923

[bib57] Woodson, G. E. (2005). Spasmodic dysphonia: Etiology and management. Perspectives on Voice and Voice Disorders, 15(1), 15–19. 10.1044/vvd15.1.15

[bib59] Woodson, G. E., Zwirner, P., Murry, T., & Swenson, M. R. (1992). Functional assessment of patients with spasmodic dysphonia. Journal of Voice, 6(4), 338–343. 10.1016/S0892-1997(05)80030-X

