# Supplementary material for: Comparing the Effects of Sensory Tricks on Voice Symptoms in Patients With Laryngeal Dystonia and Essential Vocal Tremor
Source: J Speech Lang Hear Res. 2025 Feb 27;68(4):1654–75. doi: 10.1044/2024_JSLHR-24-00476 (PMC12381841; doi:10.1044/2024_JSLHR-24-00476)
Supplement: Supplemental Material S1 [file JSLHR-68-1654-s001.pdf]

Supplemental material, Dwenger et al., “Comparing the Effects of Sensory Tricks on Voice Symptoms in Patients With Laryngeal Dystonia and Essential Vocal Tremor,” *JSLHR*, https://doi.org/10.1044/2024_JSLHR-24-00476

Supplemental Material S1. Descriptive statistics for the normal control group stratified by outcome measurement and condition.

Normal Control (N=5)

	Listener Ratings				Avg CPPS				Vocal Effort			
	Obs.	Mean ± SD	Median	Min, Max	Obs.	Mean ± SD	Median	Min, Max	Obs.	Mean ± SD	Median	Min, Max
Control 1												
Sustained Phonation	Referent for paired comparison paradigm				10	16.9 ± 2.4	18.1	12.1, 19.3	5	13.1 ± 21.3	0	0, 49
Voice loaded					29	11.8 ± 2.3	11.6	7.7, 15.3				
Voiceless loaded					29	8.7 ± 1.5	8.4	6.2, 11.5				
Control 2												
Sustained Phonation	65	1.5 ± 20	0	-54, 50	10	17.3 ± 2.4	17.3	13.6, 21.5	5	11.2 ± 25	0	0, 56
Voice loaded	96	-3.6 ± 13.4	0	-46, 50	30	11.7 ± 2.2	11.2	8.1, 15.3				
Voiceless loaded	93	-0.2 ± 16.3	0	-46, 46	30	8.5 ± 1.2	8.4	6.2, 11				
DAF												
Sustained Phonation	65	3.4 ± 19.1	0	-64, 54	10	17.3 ± 2.8	17.5	12.9, 21.7	5	17.4 ± 16.6	23.5	0, 36.5
Voice loaded	96	-9.5 ± 19.1	0	-70, 30	30	12.4 ± 2.2	12.3	8.8, 15.9				
Voiceless loaded	93	-8.5 ± 20.4	0	-94, 42	30	9.7 ± 1.4	9.4	7.6, 12.7				
VTS												
Sustained Phonation	62	-4.7 ± 22.4	0	-54, 46	10	15.3 ± 1.8	15.1	13, 18.5	5	20 ± 19.3	23	0, 39.5
Voice loaded	93	-0.6 ± 11.0	0	-38, 38	30	10.6 ± 1.8	10.4	7, 14				
Voiceless loaded	93	-1.2 ± 15.2	0	-48, 46	30	8.0 ± 1.0	8.0	6.2, 9.8				
ENDO - A												
Sustained Phonation	65	-2.1 ± 20.7	0	-50, 46	10	15.4 ± 1.3	15.3	13.3, 18.1	5	16.7 ± 17	16.5	0, 37.5
Voice loaded	93	-0.04 ± 15.2	0	-44, 56	30	11.8 ± 2.1	11.5	8.6, 15.1				
Voiceless loaded	93	-2.2 ± 17.5	0	-44, 68	30	8.8 ± 1.5	8.7	6.6, 12.7				
ENDO + A												
Sustained Phonation	62	-2.6 ± 25.9	0	-90, 46	10	16.1 ± 2.8	16.6	11.4, 20.5	5	14.9 ± 10.9	15.5	0, 26
Voice loaded	93	-4.5 ± 15.3	0	-74, 40	30	11.7 ± 2.3	10.8	7.8, 15.4				
Voiceless loaded	96	-5.4 ± 18.0	0	-56, 54	30	8.6 ± 1.4	8.4	6.4, 11.5				
