# Supplementary material for: Comparing the Effects of Sensory Tricks on Voice Symptoms in Patients With Laryngeal Dystonia and Essential Vocal Tremor
Source: J Speech Lang Hear Res. 2025 Feb 27;68(4):1654–75. doi: 10.1044/2024_JSLHR-24-00476 (PMC12381841; doi:10.1044/2024_JSLHR-24-00476)
Supplement: Supplemental Material S2 [file JSLHR-68-1654-s002.pdf]

Supplemental material, Dwenger et al., “Comparing the Effects of Sensory Tricks on Voice Symptoms in Patients With Laryngeal Dystonia and Essential Vocal Tremor,” *JSLHR*, https://doi.org/10.1044/2024_JSLHR-24-00476

Supplemental Material S2. Descriptive statistics for the laryngeal dystonia group stratified by outcome measurement and condition.

Laryngeal Dystonia (N=5)

	**Listener Ratings**				**Avg CPPS**				**Vocal Effort**			
	Obs.	Mean ± SD	Median	Min, Max	Obs.	Mean ± SD	Median	Min, Max	Obs.	Mean ± SD	Median	Min, Max
Control 1												
Sustained Phonation					10	15.9 ± 3.4	16.6	10.3, 20.4				
Voice loaded	Referent for paired comparison paradigm				30	9.3 ± 3.1	9.6	4.2, 14.3	5	54.2 ± 27.1	59.5	16, 84
Voiceless loaded					30	7.1 ± 2.5	6.8	3.5, 13.9				
Control 2												
Sustained Phonation	62	-8.2 ± 38.2	0	-100, 46	10	15.5 ± 3.1	16.4	9.9, 20.1				
Voice loaded	93	-8.7 ± 29.0	0	-82, 86	30	9.1 ± 3.1	9.3	4.3, 16	5	35.5 ± 30.7	26.5	0.5, 69
Voiceless loaded	93	-6.7 ± 32.2	0	-100, 68	30	6.6 ± 1.9	6.6	3.3, 10.1				
DAF												
Sustained Phonation	62	-8.2 ± 41.2	0	-100, 78	10	15.8 ± 3.2	15.8	11.3, 19.8				
Voice loaded	93	-21.9 ± 37.7	-24	-100, 78	30	10.7 ± 3.0	11.2	4.8, 16.3	5	55.4 ± 28.8	54	23.5, 100
Voiceless loaded	93	-23.1 ± 38.0	-26	-100, 72	30	8.8 ± 2.5	9.3	4.6, 13.2				
VTS												
Sustained Phonation	65	-21.5 ± 36.4	-20	-98, 50	10	12.1 ± 2.2	12.6	6.2, 13.8				
Voice loaded	96	-13.5 ± 37.0	0	-100, 80	30	7.6 ± 2.2	7.4	4.7, 15.7	5	48.6 ± 32.1	58.5	2.5, 84
Voiceless loaded	93	-13.1 ± 23.7	0	-86, 40	30	6.5 ± 1.9	5.9	4.2, 11.1				
ENDO - A												
Sustained Phonation	48	-11.5 ± 32.0	0	-98, 54	8	15.3 ± 4.2	16.2	9.4, 19.9				
Voice loaded	75	2.1 ± 28.0	0	-60, 72	24	9.1 ± 3.3	9.1	3.7, 16.8	4	50.1 ± 25.6	45.8	27.5, 81.5
Voiceless loaded	72	4.9 ± 22.6	0	-46, 54	24	7.3 ± 3.0	6.7	3.4, 13.1				
ENDO + A												
Sustained Phonation	62	-5.0 ±35.2	0	-92, 72	10	15.5 ± 3.4	16.6	10.3, 20.4				
Voice loaded	99	-4.1 ± 29.9	0	-72, 78	30	9.4 ± 2.7	9.8	4.6, 15.4	5	37.6 ± 28.6	22	15.5, 84
Voiceless loaded	93	-6.8 ± 23.9	0	-82, 58	30	7.6 ± 2.5	9.3	4.6, 13.2				
