# Supplementary material for: Comparing the Effects of Sensory Tricks on Voice Symptoms in Patients With Laryngeal Dystonia and Essential Vocal Tremor
Source: J Speech Lang Hear Res. 2025 Feb 27;68(4):1654–75. doi: 10.1044/2024_JSLHR-24-00476 (PMC12381841; doi:10.1044/2024_JSLHR-24-00476)
Supplement: Supplemental Material S3 [file JSLHR-68-1654-s003.pdf]

Supplemental material, Dwenger et al., “Comparing the Effects of Sensory Tricks on Voice Symptoms in Patients With Laryngeal Dystonia and Essential Vocal Tremor,” *JSLHR*, https://doi.org/10.1044/2024_JSLHR-24-00476

Supplemental Material S3. Descriptive statistics for the essential vocal tremor group stratified by outcome measurement and condition.

Essential Vocal Tremor (N=5)

	**Listener Ratings**				**Avg CPPS**				**Vocal Effort**			
	Obs.	Mean ± SD	Median	Min, Max	Obs.	Mean ± SD	Median	Min, Max	Obs.	Mean ± SD	Median	Min, Max
Control 1												
Sustained Phonation					10	14.9 ± 1.3	14.6	12.9, 17.3				
Voice loaded	Referent for paired comparison paradigm				30	9.0 ± 2.1	9.2	5.1, 13.3	5	33.8 ± 22.1	39	0, 61
Voiceless loaded					30	6.9 ± 1.1	7.0	5, 9.1				
Control 2												
Sustained Phonation	70	-1.4 ± 34.9	0	-78, 92	10	14.6 ± 3.0	15.3	10.4, 18.2				
Voice loaded	105	3.5 ± 33.9	0	-100, 82	30	8.9 ± 2.0	9.2	5.2, 12.1	5	26.1 ± 26.4	17	5, 70
Voiceless loaded	105	-1.0 ± 25.6	0	-50, 62	30	6.8 ± 1.1	6.8	4.7, 8.5				
DAF												
Sustained Phonation	70	-2.1 ± 36.0	0	-80, 84	10	14.0 ± 2.0	13.7	11.8, 18.2				
Voice loaded	105	-29.5 ± 31.5	-36	-88, 62	30	9.8 ± 2.6	9.1	6.4, 17.2	5	29.3 ± 26.8	19	0, 70
Voiceless loaded	105	-22.6 ± 28.1	-26	-72, 94	30	8.1 ± 1.5	8.0	6, 11.4				
VTS												
Sustained Phonation	70	-10.6 ± 30.3	0	-86, 54	10	10.4 ± 3.7	10.6	4.3, 16.1				
Voice loaded	105	-8.5 ± 34.8	0	-94, 80	30	7.7 ± 1.8	7.7	5.1, 11.5	5	24 ± 19.4	18.5	0, 46.5
Voiceless loaded	105	-8.2 ± 31.4	0	-78, 74	30	6.1 ± 1.1	6.2	4.5, 7.8				
ENDO - A												
Sustained Phonation	70	-8.1 ± 30.3	0	-70, 50	8	13.9 ± 2.3	14.1	10.5, 18.1				
Voice loaded	105	-17.8 ± 32.0	-16	-84, 76	24	9.8 ± 2.8	9.9	5.1, 14.3	5	28.9 ± 22.4	22.5	2.5, 61
Voiceless loaded	105	-10.5 ± 26.6	0	-100, 44	24	7.4 ± 1.6	7.7	4.8, 10.5				
ENDO + A												
Sustained Phonation	70	-8.0 ± 35.9	-7	-92, 66	10	13.0 ± 1.9	12.9	10.2, 15.4				
Voice loaded	105	-11.0 ± 29.7	-2	-88 ± 82	30	8.9 ± 1.9	9.1	5.6, 12.7	5	29.6 ± 26.8	20.5	0, 70.5
Voiceless loaded	105	-5.6 ± 24.8	0	-80, 56	30	6.9 ± 1.3	6.8	4.7, 9.2				
